# Influence of orotracheal intubation, shock and acute renal failure of elderly patients admitted to ICU

**DOI:** 10.1186/2197-425X-3-S1-A658

**Published:** 2015-10-01

**Authors:** JE Romo Gonzales, J Silva Obregón, C Martin Dal Gesso, P Gallardo Culebradas, S Saboya Sanchez, M Torralba

**Affiliations:** Hospital Universitario de Guadalajara, Intensive Care Unit, Guadalajara, Spain; Hospital Universitario de Guadalajara, Anaesthesiology, Guadalajara, Spain; Hospital Universitario Puerta de Hierro Majadahonda, Intensive Care Unit, Madrid, Spain; Hospital Universitario de Guadalajara, Internal Medicine, Guadalajara, Spain

## Objectives

Describe the clinic-epidemiological characteristics of elderly patients (³80 years) admitted to ICU for medical reasons, analyze the impact of orotracheal intubation (IOT), acute renal failure (RF) and shock at the mortality adjusted for habitual scales (APACHE II, SAPS II and SOFA).

## Methods

Retrospective cohort study from January-2003 to May-2011. In this study we included patients (³ 80 years) admitted to the ICU for medical causes, except ischemic heart disease or arrhythmias. Logistic regressions were used to calculate the OR of different variables as predictors of mortality (dependent variable). The ability to predict mortality of different "scores" adjusted for IOT, RF and Shock was performed using multivariate logistic regressions.

## Results

They were analyzed 95 patients, 58% male; median age was 81 years (IQR 80-83 years). 52 patients died (54.7%), withdrawing life-sustaining therapy in 22 patients (23.2%). They were readmitted to ICU 3.3%. The median number of days in the ICU and hospital were 4 (IQR: 1.4 to 11) and 12 days (IQR: 6-25) respectively. The median APACHE II, SAPS II and SOFA were 23 (IQR 18-29), 7 (IQR: 4-10) and 49 (IQR: 40-65). 63% was intubated (60/95) for a median of 4 days (IQR: 1-13.75), died 73.3% (44/60). 65.3% presented shock (62/95), 66.1% died (41/62). 61% renal failure (58/95) and 60.3% died (35/58).

Mortality was associated in univariate analysis with IOT (OR: 9.3; IC95% 3.5-24.6; p < 0.0001), Shock (OR: 3.9; IC95% 1.6-9.6; p = 0.002)), but not with FR (OR 1.8; IC95% 0.8-4.1; p = 0.17) or age (p = 0.65); for every one point increase in score of APACHE II, SAPS II and SOFA, the increased in mortality was 16%, 32% and 13% respectively (p < 0.0001 in all three analyzes). In a conditional stepwise multivariate analysis, adjusting for FR, IOT, shock, age, sex and different "scores", only the SAPS II (OR 1.12, 95% CI 1.6-1.18; p < 0.0001) and IOT (OR 3.6, 95% CI 1.12-11.37; p = 0.031) were independent predictors of mortality.

## Conclusions

In our series, the overall mortality of elderly patients (³ 80 years) admitted to the ICU was very high (54.7%). The orotracheal intubation and SAPS II are independent variables associated to mortality patients admitted to ICU for medical cause with at least 80 years.

